# Sex differences of the lipid profile, impulsivity and suicidality in psychiatric inpatients

**DOI:** 10.3389/fpsyt.2025.1595783

**Published:** 2025-05-19

**Authors:** Evanthia Bella, Vasilios Kafetzopoulos, Andreas Chatzittofis

**Affiliations:** ^1^ Medical School, University of Cyprus, Nicosia, Cyprus; ^2^ Department of Clinical Sciences/Psychiatry, Umeå University, Umeå, Sweden

**Keywords:** impulsivity, suicidality, suicide, HDL, LDL, lipids, inpatients, sex differences

## Abstract

**Introduction:**

Effective management of suicidality and impulsivity in hospitalized psychiatric patients is vital for improving outcomes and ensuring safety. Psychiatric patients, especially those with schizophrenia, exhibit higher tendencies towards aggressive and suicidal behaviours. This study aims to explore sex-specific associations between lipid profiles, impulsivity, and suicidality among psychiatric inpatients.

**Methods:**

A total of 158 psychiatric inpatients (92 men and 66 women) were assessed using the Barratt Impulsiveness Scale, the Columbia Suicide Severity Rating Scale, and the Karolinska Interpersonal Violence Scale. Serum lipid levels (total cholesterol, LDL, HDL) were obtained from fasting blood samples.

**Results:**

Among men, higher total cholesterol and LDL were positively correlated with impulsivity (LDL and BIS-11 total score: rho = .308, p = .006). In women, higher HDL was associated with lower suicidality (HDL and lifetime suicide attempt frequency: rho = –.374, p = .021). Regression confirmed LDL predicts impulsivity in men (R squared = .265, p = .031), and HDL, LDL, age, and BMI explain 52 percent of suicidality variance in women (R squared = .523, p = .0006).

**Conclusion:**

Elevated LDL may indicate higher impulsivity in men, while low HDL suggests higher suicide risk in women. Lipid monitoring could enhance risk assessment in psychiatric care.

## Introduction

Impulsivity and suicidality are major concerns among psychiatric inpatients, and both phenomena have been linked to an elevated risk of harm to oneself or others (1). The factors that contribute to impulsive behaviors and suicidal actions are complex, involving neurobiological, psychological, and social components (2). Recent research has highlighted associations between metabolic indicators, such as lipid levels, and high-risk behaviors, suggesting their potential as biomarkers linked to underlying biological processes (3, 4).

Abnormalities in the lipid profile, particularly involving cholesterol subfractions, have been suggested to correlate with aggression, violent behavior, and self-injurious actions in specific clinical populations (5, 6). Low total cholesterol has been reported in individuals who exhibit severe depressive symptoms, heightened impulsivity, and suicidality (7–11). Several studies suggest that low total cholesterol is related to a higher risk of suicidal behavior, especially in violent or impulsive attempts (12, 13).

In violent suicide attempters, investigations have also revealed reduced platelet serotonin and cholesterol concentrations, hinting at a potential link between serotonergic dysregulation, lipid imbalance, and the propensity for more aggressive forms of self-harm (14–16). These findings are further supported by evidence that low serum cholesterol might co-occur with habitually violent tendencies, as seen in both clinical populations and offender cohorts (17, 18). One explanation points to disruptions in serotonin metabolism, where reduced cholesterol in neuronal membranes may alter the function of serotonin receptors and transporters, potentially exacerbating impulsive or aggressive tendencies (19, 20). This hypothesis is supported by reports of lower serum cholesterol among individuals with a history of violent suicide attempts, as opposed to non-violent attempts (14, 15, 21, 22). Furthermore, large-scale population studies have found that reduced lipid levels might be linked to overall increases in both self-harm and externalizing behaviors (23, 24).

On the other hand, some authors argue that the relationship between low cholesterol and suicidality is neither universally observed nor straightforward, noting that additional factors can influence both lipid levels and psychiatric outcomes (25–27). Factors such as age, psychiatric diagnoses, and comorbid medical conditions may modulate the influence of lipids on behavior (28–30). For example, certain data suggest that higher cholesterol may also be associated with suicidal tendencies in specific subgroups only, adding complexity to the discussion (31, 32).

Sex in particular is emerging as an important factor in psychiatric disorders and their treatment (33, 34). Yet, relatively few studies have examined how sex might influence the relationship between lipid profiles and risk-taking or self-harm behaviors, despite indications of divergent lipid-risk relationships between men and women (15, 35). Biological differences, including hormonal regulation, could affect how lipids are metabolized and how they interact with neural circuits linked to impulsive decision-making (5). Women who attempt suicide have been reported to have lower serum cholesterol levels (36). Hormonal fluctuations in women may make them more susceptible to mood dysregulation, which in turn could intensify suicidal thoughts under certain metabolic conditions (8, 37). Conversely, men might exhibit alternative lipid-mediated pathways that contribute to impulsive or aggressive behavior, potentially leading to higher rates of lethal self-harm (11). Furthermore, lifestyle and psychosocial factors, such as stressors or cultural norms, could potentially differ between men and women, possibly influencing how lipid levels are associated with risk-taking or self-harm behaviors. Although some evidence suggests sex-specific mechanisms (38), further research is needed to clarify these relationships (39–41).

The primary aim of this study is to investigate sex-specific associations between lipid profiles (total cholesterol, LDL, HDL, and triglycerides) and impulsivity and suicidality among psychiatric inpatients. We hypothesize that lipid dysregulation is differentially associated with impulsivity and suicidal behaviors in men and women. Specifically, we predict that elevated LDL cholesterol levels are associated with higher impulsivity primarily among men, whereas lower HDL cholesterol levels correlate with increased suicidality predominantly among women. Our findings, interpreted considering existing literature, seek to provide new insights into the multifaceted roles that lipids may play in psychiatric conditions marked by elevated risk of self-harm or aggression (42, 43).

## Methods

### Study settings

The study participants were recruited among inpatients at Nicosia General Hospital Psychiatric Clinic and Athalassa Psychiatric hospital in Cyprus between the years 2019-2024. The patients were asked to take part in a study on biological markers focusing on suicidal and violent behavior. The study protocols (Dnr EEBK/EΠ/2019/15) were approved by the Cyprus Bioethics committee, and all patients gave their written informed consent before inclusion in the study.

### Subjects

The cohort study consists of 158 patients. There were 92 men with a mean age of 40.3 years (SD: 12.7, range: 19-70) and 66 women, mean age of 41.9 years (SD: 12.7, range: 19-67). To be included in the study, all participants must exhibit a fair capacity to communicate verbally and in writing the Greek language and be 18 years or older. Psychiatric inpatients with diagnoses of mood disorder (depression, bipolar disorder), psychosis spectrum disorder (schizoaffective disorder, schizophrenia, delusional disorder), personality disorders and anxiety related disorders were included in the study.

Exclusion criteria were inability to give informed consent to participate in the study, ed psychiatrist and diagnosis was established according to DSM-5 criteria. Further information was acquired from the medical file of the patient and the multidisciplinary treatment team of the clinic. Trained clinicians performed and assessed all interviews and ratings.

In this study 43% of the patients fulfilled criteria for having schizophrenia, 24% for mood disorders and 5% for anxiety disorder. The subjects were generally somatically healthy. The non-psychiatric diagnoses of the patients at study inclusion were metabolic syndrome and diabetes, chronic obstructive pulmonary disease, hypertension, glaucoma and hypo/hyperthyroidism.

### Serum cholesterol assays

Blood samples were collected under controlled conditions from the antecubital vein after participants had fasted overnight, which helps ensure more accurate measurements by minimizing the influence of recent dietary intake. The samples were then processed at the Laboratory of Clinical Chemistry in the Nicosia General Hospital, where total serum cholesterol, LDL cholesterol, and HDL cholesterol were measured using standard laboratory procedures.

### Assessments

The Barratt Impulsiveness Scale (BIS-11) (44, 45) is a 30-item self-report questionnaire designed to evaluate impulsive levels in both clinical and non-clinical populations. Respondents rate each item on a four-point Likert scale, from 1 (Rarely/Never) to 4 (Almost Always/Always), producing total scores that can range from 30 to 120. Higher scores correspond to greater degrees of impulsiveness. The BIS-11 organizes impulsivity into six first-order factors—attention, motor, self-control, cognitive complexity, perseverance, and cognitive instability—and three second-order factors (cognitive, motor, and non-planning impulsiveness). This multidimensional structure helps clinicians and researchers identify specific areas of impulsivity, which can be particularly important for tailoring interventions in conditions where impulsive behavior plays a significant role.

The Columbia–Suicide Severity Rating Scale (C-SSRS) (46, 47) is a standardized measure commonly used in both clinical and research settings to assess the presence and intensity of suicidal ideation, as well as suicidal behaviors. It captures critical details such as the frequency of suicidal thoughts and any history of suicide attempts over a person’s lifetime or within a specified timeframe (e.g., the past three months). By evaluating these factors, clinicians and researchers can identify individuals at higher risk, monitor changes in risk level over time, and make informed decisions about the most appropriate intervention strategies. In this context, data on suicide attempts, lifetime and recent suicidal behavior, and the frequency of suicidal thoughts were specifically analyzed to provide a clearer picture of the individual’s overall risk profile.

The Karolinska Interpersonal Violence scale (KIVS) consists of four subscales, designed to assess the exposure to and expression of interpersonal violence as a child (between 6 and 14 years of age), and as an adult (15 and older), (48). The ratings are based on a semi-structured interview and items refer to serious events during the lifetime. The scoring is between 0 and 5 for all four subscales (49, 50).

### Data analysis

To establish the reliability of the tests used, we measured Cronbach’s α for the BIS and C-SSRS scale. We did not use this metric for KIVS as each item is assessed independently. We used Spearman’s rank correlation coefficient (Spearman’s ρ) to investigate relationships among our variables because it does not require data to observe a normal distribution—an important consideration given that most of our groups did not meet normality criteria. After identifying significant correlations, we applied a linear regression model to further explore these associations, using a stepwise approach to systematically introduce additional variables while ensuring the model’s best fit. The assumption of the regression analysis, specifically linearity of the relationship, homoscedasticity using the Breusch-Pagan test, normality of residuals using a Shapiro-Wilk test and the absence of overly influential outliers calculating Cook’s distance for each data point were tested and met. Throughout all analyses, we employed a 95% confidence level (α = 0.95) to determine statistical significance, and we presented the data in graphs as mean ± standard error of the mean (SEM), mean and standard deviation in tables, and Spearman rho values with a heatmap in correlational matrices. This methodological approach helps ensure that our results accurately capture the relationships between variables while accommodating the distributional characteristics of our data. Data analyses were performed using R (51, version 4.2.0), which provided a robust environment for statistical computing. Custom scripts were developed in Python (52, version 3.9) to handle data processing and visualization.

## Results

### Sample

Our sample consists of 158 inpatients, 92 men and 66 women. In the BIS questionnaire, the sample characteristics were (mean = 68.03, SD = 13.29, range = 41 – 112; mean = 69.01, SD = 13.19, range = 43 – 112; mean = 66.55, SD = 13.47, range = 41–102 for the total sample, men, and women respectively). The Cronbach’s α of the BIS scale was 0.81 (95% CI 0.77 - 0.85).

In C-SSRS, for suicide attempts throughout the lifetime, the sample characteristics were (mean = 1.66, SD = 2.15, range = 0 – 5; mean = 1.32, SD = 2.00, range = 0 – 5; mean = 2.15, SD = 2.27, range = 0–5 for the total sample, men, and women respectively). Accordingly, in suicide attempts in the last 3 months, our sample was as follows (mean = 1.66, SD = 5.00, range = 0 – 50; mean = 1.53, SD = 6.00, range = 0 – 50; mean = 1.85, SD = 3.30, range = 0–16 for the total sample, men, and women respectively). The Cronbach’s α of the C-SSRS scale was 0.9 (95% CI 0.87 - 0.92).

When it came to the KIVS scale, use of violence in adulthood was measured, and the sample characteristics were (mean = 1.22, SD = 1.44, range = 0 – 5; mean = 1.53, SD = 1.51, range = 0 – 5; mean = 0.78, SD = 1.23, range = 0–4 for the total sample, men, and women respectively). In victim of violence in childhood, the sample characteristics were (mean = 2.10, SD = 1.72, range = 0 – 5; mean = 2.21, SD = 1.56, range = 0 – 5; mean = 1.97, SD = 1.92, range = 0–5 for the total sample, men, and women respectively).

For clarity, each value we measured and used in our analyses is presented in **Supplementary Table 1** organized by test performed and sex.

### Impulsivity, lipid profile and sex differences

**Figure 1:**
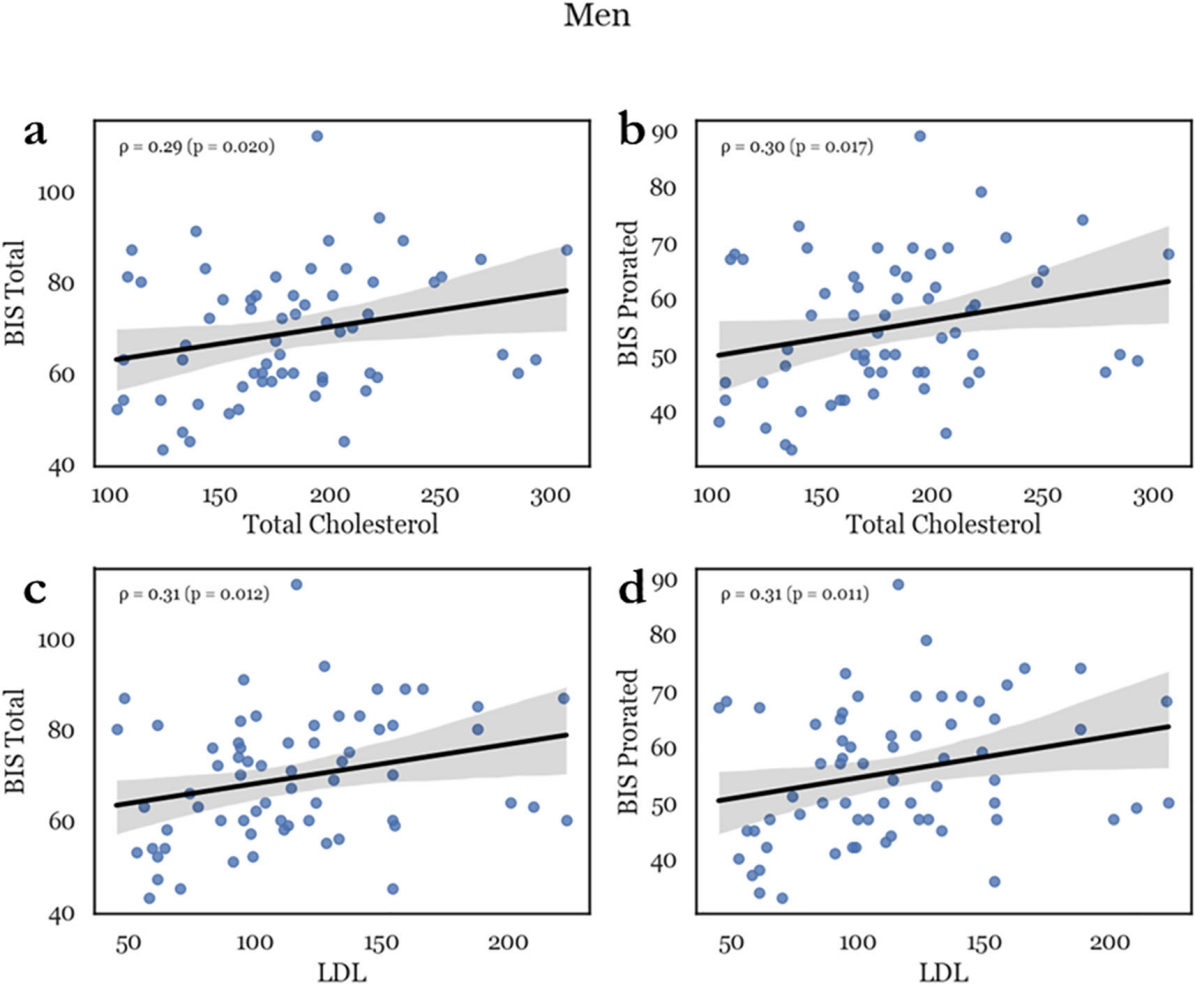
Representative scatterplots depicting the correlation between lipids and impulsivity in men. Total cholesterol (TC) was positively correlated with impulsivity scores such as total BIS score **(a)** and BIS prorating **(b)**. LDL similarly correlated with total BIS score **(c)** and prorating **(d)**, with an ρ between 250 and 300 which indicated a medium effect.

Men exhibited a pronounced positive correlation of total cholesterol with BIS Total, BIS Prorating Total, BIS Factor Non-Planning, and BIS Factor Cognitive (ρ = .293, p = .01; ρ = .299, p = .009; ρ = .238, p = .03; and ρ = .301, p = .008, respectively). Similarly, LDL levels were correlated with the same ratings (BIS Total, BIS Prorating Total, BIS Factor Non-Planning, and BIS Factor Cognitive: ρ = .308, p = .006; ρ = .310, p = .006; ρ = .241, p = .026; and ρ = .320, p = .004, respectively). (see **Figure 1**) Virtually all impulsivity factors were linked to cholesterol and LDL, except for motor impulsivity (BIS Motor: cholesterol, LDL: ρ = .158, p = .109; and ρ = .159, p = .116, respectively). However, there was no correlation between impulsivity measures and HDL in men. In women there was no association between impulsivity scales and total cholesterol, HDL or LDL (BIS total *vs* total cholesterol, HDL and LDL: ρ = -.285, p = .102; ρ = -.163, p = .343; and ρ = -.136, p = .429, respectively) (see **Supplementary Figure 1**).

### Suicidal behavior, lipid profile and sex differences

In men, there was no correlation between suicidality measures and either total cholesterol, HDL or LDL. All correlations are depicted in **Supplementary Figure 2**.

**Figure 2:**
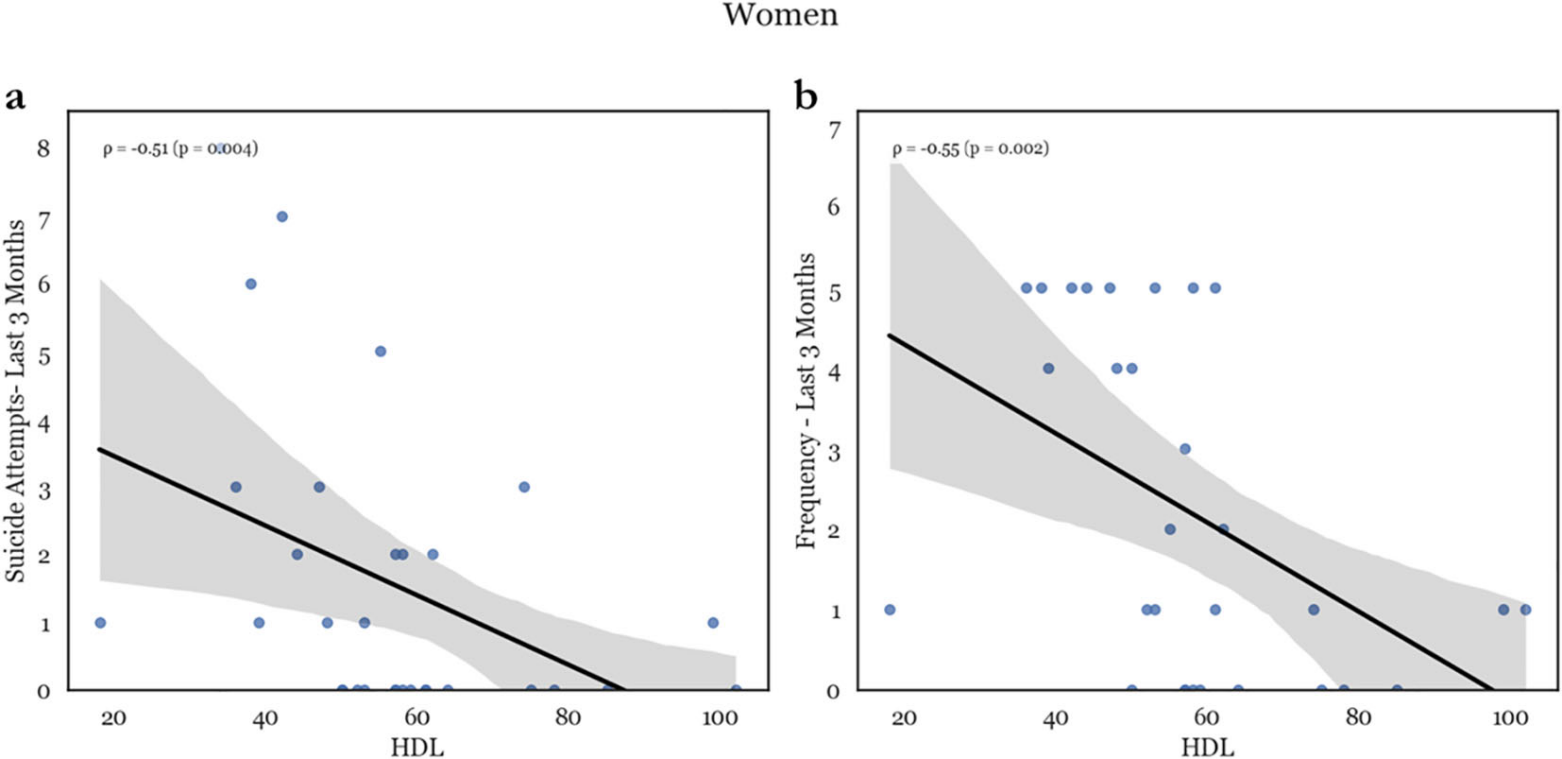
Representative scatterplots of HDL levels with suicide attempts in the past 3 months **(a)** and frequency of ideation in the past 3 months **(b)**. There is a major negative correlation between HDL levels and both recent attempts as well a recent suicidal ideation.

In women, higher cholesterol was associated with decreased lifetime suicide attempts and lifetime suicide behavior (CSSRS suicide attempts – lifetime, CSSRS suicidal behavior – lifetime: ρ = -.440, p = .01; and ρ = -.322, p = .044, respectively). (see **Figure 2**) On the other hand, HDL was negatively correlated with most measured suicidality factors (CSSRS suicide attempts – lifetime, CSSRS suicide attempts – 3-month, CSSRS suicidal behavior – lifetime, CSSRS suicidal behavior – 3-month, CSSRS suicidal ideation – 3-month, CSSRS suicidal ideation frequency – lifetime, and CSSRS suicidal ideation frequency – 3-month: ρ = -.374, p = .021; ρ = -.506, p = .002; ρ = -.314, p = .043; ρ = -.459, p = .005; ρ = -.359, p = .024; ρ = -.451, p = .005; and ρ = -.546, p <.001, respectively). Conversely, in women there was no association between suicidality and LDL. (see **Supplementary Figure 2**).

### Violence and lipids in both sexes

In the current dataset there was no statistically significant correlation between lipids and the status of either victim or perpetrator of violence, as measured by KIVS. For men, neither HDL, LDL or total cholesterol was associated with victimhood or use of violence in childhood, or victimhood or use of violence later in adult life. In women, we similarly failed to uncover a statistically significant relationship of the above measures (see **Supplementary Figure 3**).

However, KIVS significantly correlates with impulsivity in both sexes, and with suicidality in women but in men. Specifically, in men use of violence in childhood as measured in KIVS correlated closely with total impulsivity (BiS total, BiS prorated: ρ = .257, p = .018; and ρ = .260, p = .0.16, respectively) and so did status of victimhood of violence in childhood (BiS total, BiS prorated: ρ = .297, p = .006; and ρ = .261, p = .0.16, respectively). No measures in CSSRS correlate with KIVS values.

In women, impulsivity as measured by BIS is interestingly more closely correlated with use of violence in childhood (BIS total, BIS prorated: ρ = .358, p = .006; and ρ = .383, p = .0.003, respectively) and victimhood of violence in childhood (BIS total, BIS prorated: ρ = .356, p = .006; and ρ = .341, p = .0.009, respectively) as measured by KIVS. However, these very same items of the KIVS scale correlate with current suicide ideation and number of attempts throughout the lifetime (KIVS use of violence in childhood *vs* CSSRS suicidal ideation – 3-month, CSSRS suicide attempts – lifetime: ρ = .266, p = .047; and ρ = .274, p = .043., respectively, KIVS victimhood of violence in adulthood *vs* CSSRS suicidal ideation – 3-month, CSSRS suicide attempts – lifetime: ρ = .302, p = .021; and ρ = .340, p = .0.11., respectively, and KIVS victimhood of violence in childhood *vs* CSSRS suicide attempts – lifetime: ρ = .276, p = .011). This three-way overlap but not a direct link of correlations with lipid profiles over both sexes might point to a deeper causal link that has yet to be uncovered between trauma, impulsivity and suicidality (see **Supplementary Figure 3**).

### Regression analyses

To further investigate our findings, we applied linear regression models separately for men and women to validate the previously observed correlations between impulsivity and suicidality.

Subsequently, we constructed a regression model based on the results of our correlational analysis, introducing variables in a stepwise manner to identify the optimal model. The Barratt Impulsiveness Scale (BIS) score served as the dependent variable. The best-fitting model included LDL cholesterol as the sole independent variable. This model was statistically significant (F_(1,64)_ = 4.824, p = 0.031) with an adjusted R² of 0.265, indicating that the linear regression analysis confirmed the results suggested by the correlational analysis.

In the female cohort, we conducted a similar linear regression analysis but were able to include more variables in the model. We began by introducing HDL cholesterol, LDL cholesterol, triglycerides and total cholesterol stepwise with suicidal ideation during the past month as the dependent variable. The objective was to explore the previously observed relationship between HDL cholesterol and suicidality and to develop a model that could explain the relationship between blood lipid concentrations and suicidal behavior. Total cholesterol was excluded as a predictor due to significant collinearity with LDL (VIF = 13.26). The best fit model included the above variables as well as age and BMI as covariates, while triglycerides were excluded from the final analysis as they did not meet the significance threshold (p < 0.05).

The final model was statistically significant (F_(4,23)_ = 5.697, p = 0.006) with an adjusted R² of 0.5225. This model suggests that the above variables together account for approximately 52% of the variance in suicidality among hospitalized women.

## Discussion

Understanding the intricate relationship between cholesterol levels, impulsivity, and suicidality in hospitalized patients can provide valuable insights into mental health and inform targeted treatment strategies. Our findings, combined with a growing body of literature, highlight the need to consider not only conventional cardiovascular factors but also how lipid dysregulation may interact with neurobiological and psychosocial variables to influence behavior and mood.

### Low-density lipoprotein

A major focus of this investigation was on LDL cholesterol—often referred to as “bad” cholesterol due to its association with cardiovascular risk (53)—and its nuanced role in brain function. Elevated LDL levels have been linked to changes in membrane fluidity and neurotransmitter receptor functioning, particularly in regions implicated in impulse control, such as the prefrontal cortex (54). Higher LDL may, therefore, contribute to heightened impulsivity by disrupting serotoninergic and dopaminergic signaling pathways that regulate mood and behavioral inhibition. This phenomenon may help explain observed links between elevated LDL levels and increased impulsivity, especially among men.

Cholesterol’s potential impact on aggression and the nature of suicidal behavior remains complex (13). Previous studies have suggested that alterations in lipid profiles could be more closely linked to violent rather than non-violent forms of suicide, potentially due to the interplay between aggression and dysregulated neurobiological pathways (55). However, in our sample, we found no significant relationship between LDL (or high-density lipoprotein [HDL]) levels and suicidality in men, given the assumptions involved in a correlation analysis. This suggests that while LDL may contribute to impulsivity, suicidality may depend more on a constellation of factors, including genetic predispositions, psychosocial stressors, or comorbid mental health conditions (56).

### High-density lipoprotein

Another critical component of lipid dysregulation relates to HDL cholesterol, often termed “good” cholesterol for its cardiovascular protective effects (57). Beyond heart health, HDL may exert neuroprotective influences by mitigating oxidative stress and inflammation—both implicated in depressive symptoms and suicidal ideation (58). In our findings, lower HDL levels in women were associated with higher suicidality. While the nature of our study precludes causal interpretations, this association raises the possibility that HDL may play a role in pathways related to mood regulation and stress response, warranting further investigation in longitudinal research. Further, inflammation or endocrine dysregulation, which can be exacerbated by abnormal lipid profiles, has been increasingly recognized as a factor in the pathogenesis of both depression and suicidal behavior (59, 60).

The absence of a strong link between cholesterol levels and impulsivity in women in our study and in literature (61) suggests that other mechanisms such as hormonal regulation or genetic predisposition (62) may play a larger role in influencing impulsivity. Estrogen, for instance, is known to affect mood, cognitive function, and potentially aggression and impulsivity (63).

Moreover, it is crucial to consider potential reverse causality when interpreting associations between lipid profiles and suicidality. Individuals experiencing severe mood disorders or suicidal ideation may exhibit changes in appetite and physical activity that result in weight fluctuations and altered serum lipid levels (64). Thus, while dyslipidemia could contribute to suicidality risk in women, it may also be a consequence of mental health changes, emphasizing the need for longitudinal studies to clarify causality.

### Strengths and limitations

This study has several strengths, including a well-characterized inpatient psychiatric population and a comprehensive assessment of violence, suicidality, and impulsivity. However, it also has limitations. The cross-sectional design prevents conclusions about causality, making it difficult to determine whether lipid abnormalities directly influence impulsivity and suicidality or are merely associated with them. Additionally, the relatively small sample size, especially for women, limits the ability to detect more subtle effects, increasing the risk of false-negative findings. The focus on hospitalized patients only and the absence of a matched healthy control group and missing data further constrain the interpretation of our results. Additionally, due to the primarily correlational approach of our study, our findings have interpretive barriers as causality and the influence of cofounders cannot be definitely determined. Future studies with larger, well-matched cohorts and multivariate, longitudinal designs are needed to confirm these findings.

## Conclusion

Our findings highlight the complex relationship between lipid metabolism, impulsivity, and suicidality, with distinct sex differences. Elevated LDL cholesterol may serve as a potential marker of impulsivity in men, while lower HDL levels could indicate higher suicide risk in women. These insights suggest that clinicians should consider sex-specific risk assessments, including lipid profile monitoring, lifestyle modifications, and targeted pharmacological interventions. Further research is needed to clarify the biological, genetic, and psychosocial mechanisms linking lipid dysregulation to suicidal and aggressive behaviors in diverse psychiatric populations.

## Data Availability

The raw data supporting the conclusions of this article will be made available by the authors, without undue reservation.
