# Supplementary material for: Sex differences of the lipid profile, impulsivity and suicidality in psychiatric inpatients
Source: Front Psychiatry. 2025 May 19;16:1595783. doi: 10.3389/fpsyt.2025.1595783 (PMC12128606; doi:10.3389/fpsyt.2025.1595783)
Supplement: Supplementary file 1 [file DataSheet1.docx]

**Supplementary Table 1:** Characteristics of the sample with a value of central tendency (Mean) and a measure of variation (Standard Variation) for each of the three phychometric tests (Baratt Impulsiveness Scale – BIS, Columbia Suicide Severity Rating Scale – CSSRS, Karolinska Interpersonal Violence Scale – KIVS). Data is organized for the total of the population and each of the sexes.


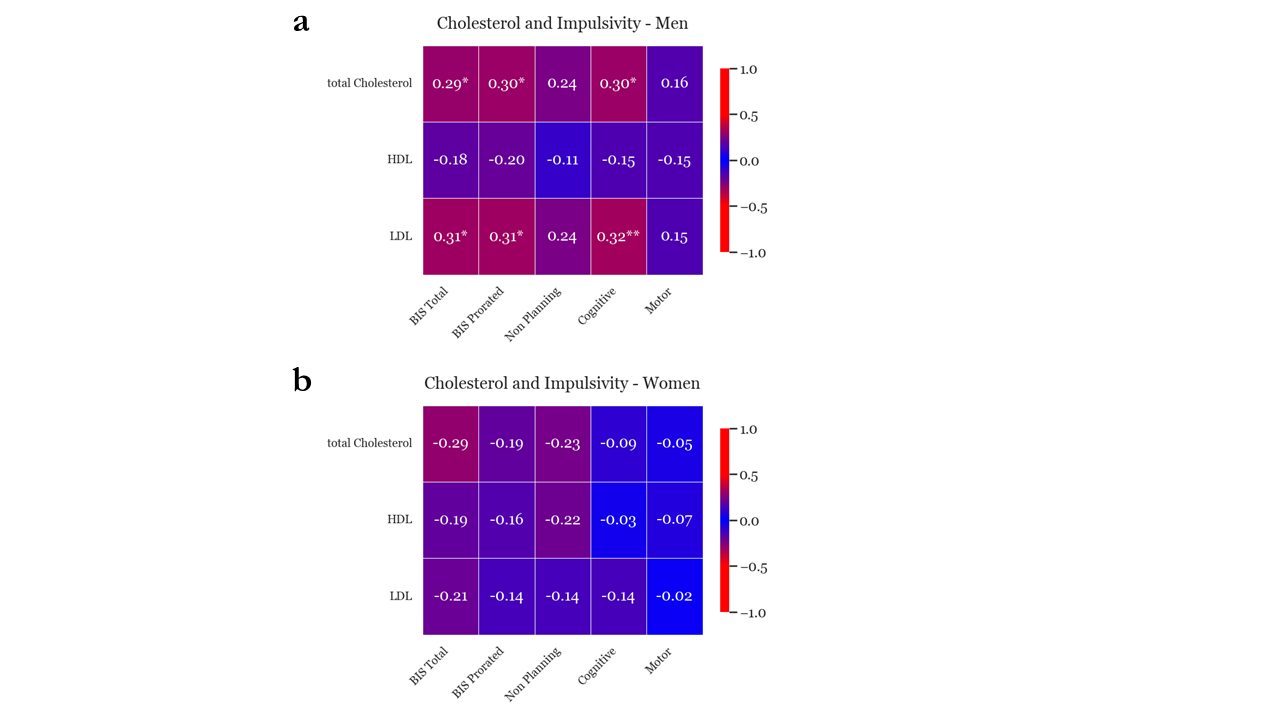


**Supplementary Figure 1**: Correlation matrix of lipids versus impulsivity as measured by the Baratt Impulsivity Scale (BIS) in men (**a**) an women (**b**). In men, lipids and especially total cholesterol and LDL were correlated with several impulsivity indices, while in women there was no significant correlation between lipid profile and impulsive behavior. (*: *p* < .05, **: *p* < .01, ***: *p* < .001)


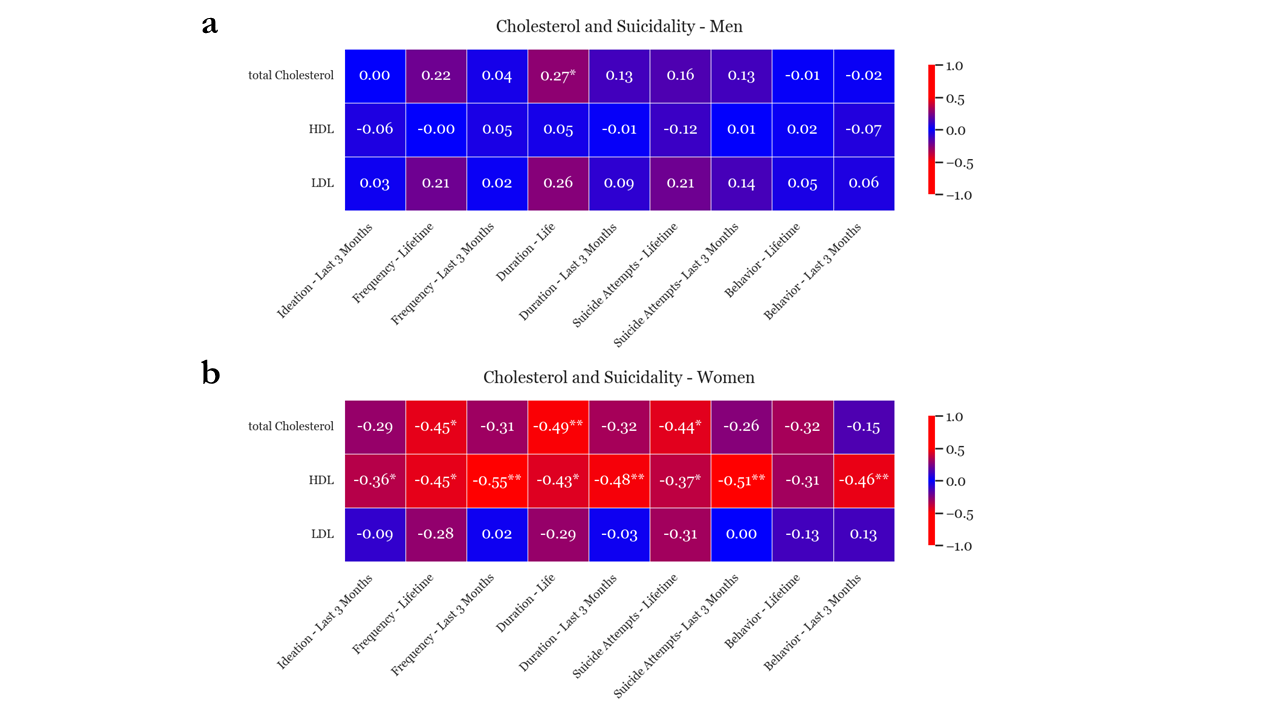


**Supplementary Figure 2**: Correlation matrix of lipids versus suicidality values as measured by the Columbia Suicide Severity Rating Scale (CSSRS) in men (**a**) and women (**b**). There was no significant correlation between the lipid profile and suicidality as measured by CSSRS in men, while in women total cholesterol and HDL was strongly correlated with a number of items from the CSSRS, emphasizing the relationship of the lipid profile with suicidality in women. (*: *p* < .05, **: *p* < .01, ***: *p* < .001)


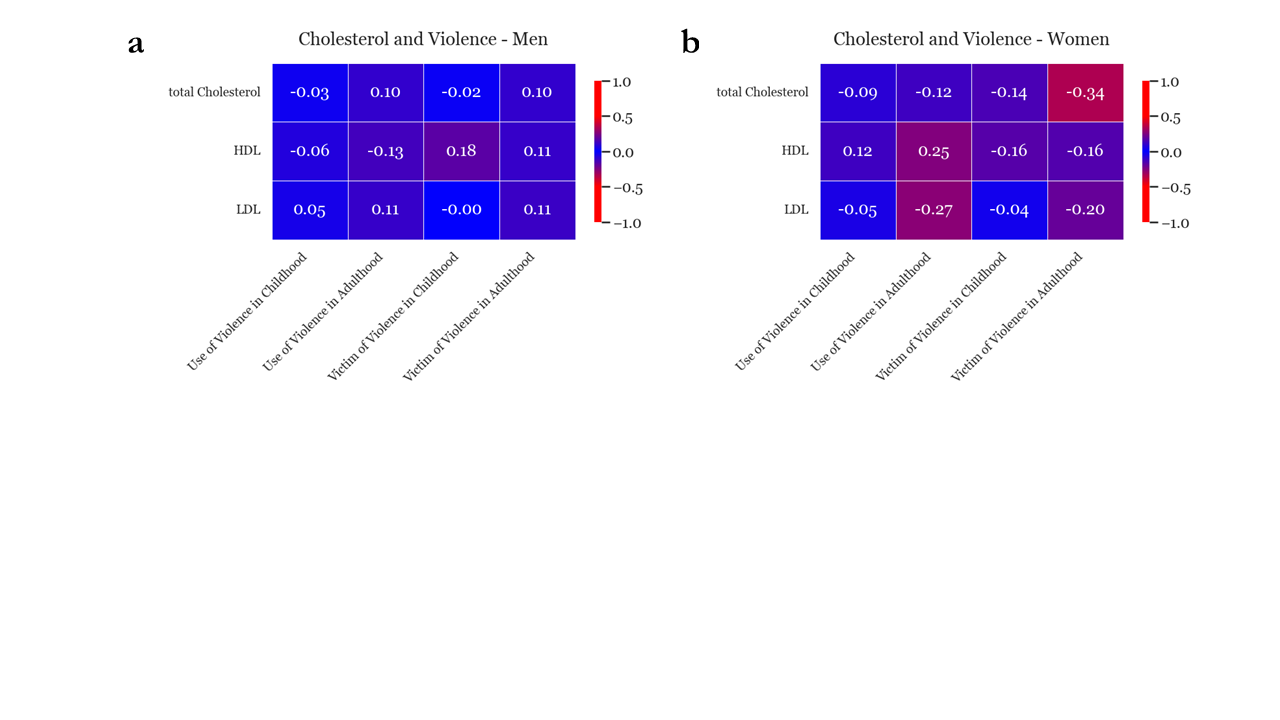


**Supplementary Figure 3**: Correlation matrix of lipids versus violence when measured by the Karolinks Interpersonal Violence Scale (KIVS) in men (**a**) an women (**b**). In both sex there was no statistically significant correlation between use and victimhood of violence and lipid profile.


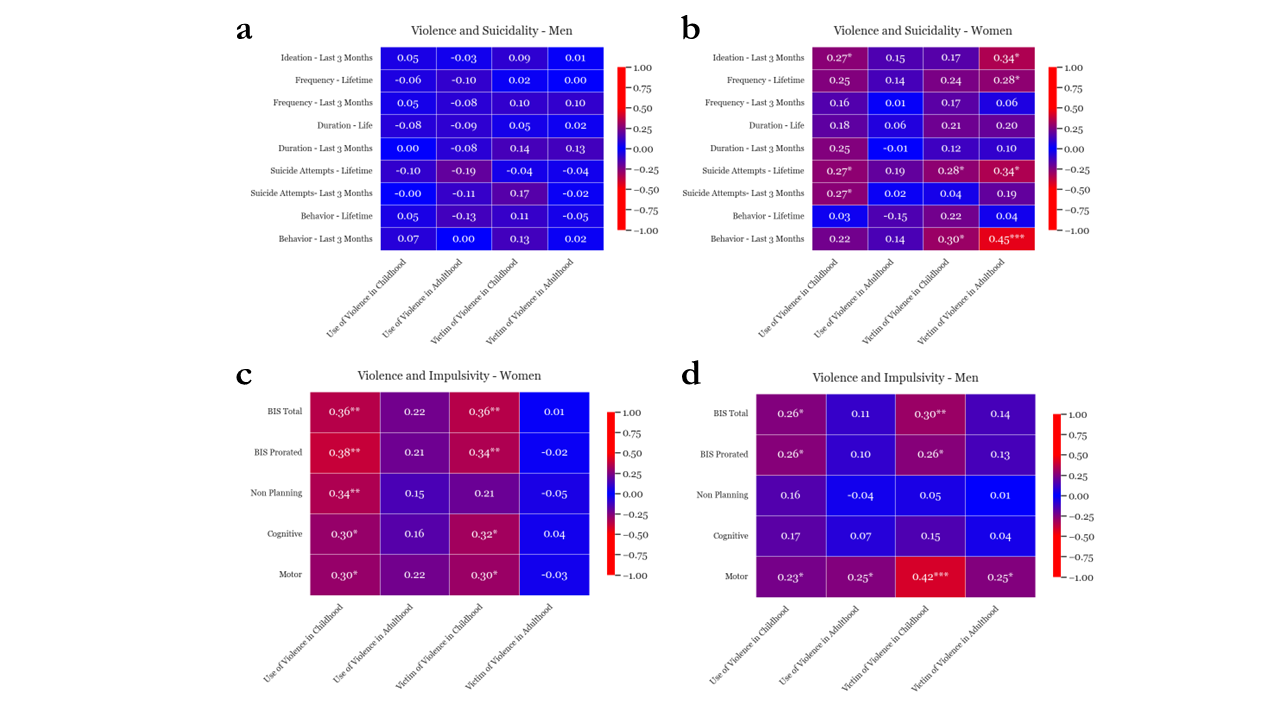


**Supplementary Figure 4**: Correlation matrices of violence and suicidality in men (a) and women (b), as well as violence versus impulsivity in men (c) and women (d). Use and victimhood of violence in childhood closely correlate with suicidality and impulsivity in women, but not in men. (*: *p* < .05, **: *p* < .01, ***: *p* < .001)
